# Editorial: The Bacterial Cell: Coupling between Growth, Nucleoid Replication, Cell Division, and Shape Volume 2

**DOI:** 10.3389/fmicb.2019.02056

**Published:** 2019-09-04

**Authors:** Ariel Amir, Jaan Männik, Conrad L. Woldringh, Arieh Zaritsky

**Affiliations:** ^1^School of Engineering and Applied Sciences, Harvard University, Cambridge, MA, United States; ^2^Department of Physics and Astronomy, University of Tennessee, Knoxville, Knoxville, TN, United States; ^3^Bacterial Cell Biology, Faculty of Science, Swammerdam Institute for Life Sciences, University of Amsterdam, Amsterdam, Netherlands; ^4^Faculty of Natural Sciences, Ben-Gurion University of the Negev, Be'er Sheva, Israel

**Keywords:** cell cycle, nucleoid, divisome, cell division, cell envelope, cell shape, chromosome replication

The bacterial cell cycle has been under intense investigation for many decades, yet many of the most fundamental questions remain wide open. DNA replication, cell division, protein synthesis, and cell envelope production are believed to be all coupled, but the causal relations between these different processes, and even which process is downstream of which, are not always known. Moreover, it is not clear to what degree the cellular “architecture” governing this coupled, complex system is conserved across different bacteria, or even within one bacterial species across different growth conditions!

Many investigations in biology focus on a small subset of proteins/other cellular components and explore how they function upon genetic or external perturbations. To study the bacterial cell cycle it is beneficial, even obligatory, to also consider the relations between various processes that *a priori* could have been thought of as completely uncoupled from each other, as for instance are transcriptional activity and nucleoid compaction. This may be the reason why this problem has been a “tough egg to crack” and why many basic questions remain unclear to date.

Recent advances in microscopy and microfluidics technologies have enabled to gather large amounts of single-cell data. Combined with mathematical modeling and statistical analyses these datasets are beginning to reveal novel and unexpected couplings between cell cycle processes. Such approaches reinvigorated the field recently and led to some exciting results, which altogether have motivated this Edition.

The contributions to this volume are divided to three Chapters: (1) Coupling between Major Cellular Processes, (2) Cell Growth and Division, and (3) Bacterial Nucleoid and Initiation of Replication. The following briefly summarizes the contributions to these three topics in that order.

The first Chapter starts with the work of Kleckner et al. that addresses the coupling between cell division and chromosome replication in *Escherichia coli*. The early studies by Cooper and Helmstetter from the 1960's have been consistent with such a coupling, where the former is downstream the latter, both being downstream of a third cellular series of processes. Here Kleckner et al. postulate a different order of couplings by extending their previous “licensing” hypothesis (referred to as a “progression permission” now). “Permission” comprises both cell growth (mass accumulation) and divisome assembly combined with placement of the terminus domain at mid-cell, which is proposed to cause a global change in nucleoid organization. Progress of this “permission” implements two parallel downstream events: replication initiation and septum closure, that occur in a comparable way in both slow and fast growth conditions.

Logsdon and Aldridge review growth and size control in mycobacteria and conclude that division is tightly linked to DNA replication, albeit in a different way than the Cooper-Helmstetter model, whereby a constant volume (rather than constant time) is added from initiation of DNA replication to division. Within the “parallel adder” model proposed, two independent volume integrators are operating simultaneously, one controlling cell division and the other initiation of DNA replication. To further complicate matters, in mycobacteria such as *M. smegmatis* division is asymmetric, and one has to distinguish between “accelerator” and “alternator” cells—each possessing a different volume increment.

Another “theme” relevant for several contributions regards the role of variability between genetically identical cells and its utility in studying cell cycle-related problems. Grilli et al. demonstrate how growth can be characterized phenomenologically by utilizing natural fluctuations (i.e., stochasticity). Correlations between key cell cycle variables are used as an additional method of characterizing phenotypes; systematic analysis of correlations between cell size and generation time in *E. coli*, for instance, constrains the landscape of possible molecular mechanisms, the details of which are yet to be understood.

In contrast, Govindarajan et al. show how isogenic cells may utilize variability to increase the population's fitness: clustering of EI, a protein important for sugar uptake and metabolism, occurs stochastically and leads to phenotypic diversity. Furthermore, it differs between growing and non-growing cells. The enhanced clustering in non-growing conditions is suggested to confer a fitness advantage, as the EI clusters provide a reservoir to be utilized at times of need.

Gangwe Nana et al. also study phenotypic variability in *E. coli*, in this case arising from asymmetric partitioning of a cellular resource between two daughter cells. The authors use Secondary Mass Spectrometry imaging to infer growth rates and observe that distribution of their ^15^N label is not homogenous in the cells but asymmetric between the cell halves. They propose that growth rate diversity relates to asymmetric partitioning of cellular resources during division, hypothesizing that inheritance of DNA strands between daughters is responsible for this asymmetry. According to the hypothesis the two parental strands of DNA are expected to be physically associated with different proteins conveying to them a different survival strategy.

Even if universal principles governing cell cycle regulation do exist in bacteria, they must operate on a specific cellular machinery, which differs from species to species. Key components of this machinery are divisome and elongasome complexes responsible for cell division and elongation, respectively. These cellular components are discussed in the second Chapter. Three review papers by van Teeseling et al., den Blaauwen, Caspi and Dekker highlight both the diversity of these machineries in different microorganisms as well as common themes. Much of the research so far has focused on a limited number of model organisms, in particular *E. coli*. Its close relative, *Thiosymbion*, divides *parallel* to the long axes of the cell, unlike the conventional model system. den Blaauwen hypothesizes that mutations in two key coordinating proteins of divisome and elangosome, FtsZ, and MreB respectively, may allow reorienting the cell division plane. Interestingly, this mode of lateral growth and longitudinal division challenges current ideas about DNA segregation and nucleoid movement.

van Teeseling et al. tackle the question of what leads to bacterial morphological diversity. While the key architecture for peptidoglycan synthesis is largely conserved in most cells, many additions to this architecture allow diversification of the morphologies leading to adaptation of the cells to their particular ecological niche. These additions, which in some cases are important for their virulence, may be useful targets for narrow band antibiotics.

Caspi and Dekker expand the topic of cell division to archaea and overview the Cdv system. They argue that although Cdv is similar to the eukaryotic ESCRT system, it is functionally different. Along the lines of common and diverging features, Flores et al. show that the placement of division in *Agrobacterium tumefaciens* depends on the widespread Min system but also on other molecular systems; the origin of which is yet to be determined.

A common theme in bacterial cell division is that septal closure needs to overcome high turgor pressure. What cellular process or structure provides the force for constriction is not clear yet. Osawa and Erickson hypothesize that among other factors excess membrane synthesis during division may force it to bleb inwards and such membrane deformation may create sufficient force to pinch the cell at its middle. Interestingly, Kumar et al., who present new experimental data on Z-ring dynamics in *E. coli*, observe that the radius of FtsZ protofilament decreases faster than the cell envelope radius. Could the difference in these two rates be explained by excess inner membrane added to the septal region as hypothesized by Osawa and Erickson?

Microfluidic techniques have become important tools in studies of growth and division. It has generally been taken for granted that bacteria grow in such devices similarly to classical liquid cultures. This has been examined by Yang et al. who show that growth rate and cell sizes in these devices are sensitively dependent on length and cross-sectional dimensions of the channels. The main growth-limiting factor in these devices is assigned to mechanical friction forces rather than nutrient limitation in the narrow channels.

A key component of cellular “architecture” mentioned above is the nucleoid, the structure and replication of which are discussed in the third Chapter of this volume. Applying Structural Illumination Microscopy, Martin et al. show that upon deletion of ribosomal RNA operons, nucleolus-like compartments are no longer formed on the nucleoid border and the nucleoid expands. They propose that long-range interactions between transcription foci may play a role in DNA compaction. Katayama et al. discuss the structural features of the *oriC* region and the regulatory cycle of DnaA-ATP, whereas Rao et al. demonstrate the influence of DnaA-ATP binding to mutated recognition sites on the timing of replication-initiation. These and other aspects of the orisome assembly have been integrated by Hansen and Atlung in their discussion of the Initiator Titration Model. Their model also shows how baby cells of different sizes that result from asymmetric divisions will initiate at different times, but will have about the same size (but not the same age) at the next initiation. The same model can also explain the larger amount of DNA in large newborn cells at fast growth as measured by Huls et al. These authors furthermore find that the larger siblings that initiate earlier segregate their nucleoids faster thus allowing them to divide earlier at a shorter length as dictated by the adder principle. In this view both initiation of replication and the processes leading to cell division are coupled to growth rate, as also emphasized by Kleckner et al.

## Author Contributions

All authors listed have made a substantial, direct and intellectual contribution to the work, and approved it for publication.

### Conflict of Interest Statement

The authors declare that the research was conducted in the absence of any commercial or financial relationships that could be construed as a potential conflict of interest.

